# 25 The Impact of Outpatient Rehabilitation Settings

**DOI:** 10.1093/jbcr/iraf019.025

**Published:** 2025-04-01

**Authors:** Dania Johnson, Trevor Pickering, Dawn Kurakazu, Jody Sabel, Karen Kowalske, Jeffrey Schneider, Haig Yenikomshian

**Affiliations:** University of California Keck School of Medicine; University of Southern California; Los Angeles General Medical Center; Harborview Medical Center, University of Washington; University of Texas Southwestern; Spaulding Rehabilitation Hospital, Harvard Medical School; University of California Keck School of Medicine

## Abstract

**Introduction:**

Burn recovery can involve extensive rehabilitation after discharge from acute care, with physical therapy (PT) and occupational therapy (OT) playing key roles in improving physical function and reintegration. Outpatient rehabilitation may occur at burn centers or external facilities without PT/OT specialists due to factors like geography and insurance. Limited data exists on how these settings impact patient-reported outcomes. This study aims to explore the settings where patients receive outpatient rehabilitation and assess differences in functional outcomes between burn centers and external facilities.

**Methods:**

This is a retrospective analysis of adult burn survivors over the age of 18 from 2016-2024 using a multicenter longitudinal patient-reported outcome database. Participants were classified as receiving rehabilitation if they endorsed PT/OT usage in the last 6 months. Service settings were categorized as either within or external to a burn center at 6, 12, and 24 months post-discharge. Demographics and usage rates were collected at all time points. Mixed-effect models, both adjusted (for demographic and insurance factors, TBSA) and unadjusted, assessed the impact of burn center PT/OT services on functional outcomes (physical function, social roles, community integration) at 6 and 12 months compared to external centers. Lagged models explored how 6-month therapy predicted 12-month outcomes.

**Results:**

Of 1,520 participants meeting inclusion criteria, 868 had data at 6 months, 679 at 12 months, and 496 at 24 months. PT/OT use peaked at 6 months (48.5%) and declined at 12 (28.6%) and 24 months (18.1%). Of these, the proportion receiving burn center care decreased over time (6 months 37.5%, 12 months 31.3%, 24 months 25.9%). In the unadjusted lagged model, burn center care at 6 months was associated with 3.33-points higher social roles at 12 months (p=.044) compared to those treated outside. The adjusted lagged model showed 1.06-point higher community integration at 12 months (p=.011) compared to those treated outside. Cross sectional analysis comparing burn center care versus outside showed no discernable effect on physical function, social roles or community integration at 6 or 12 months.

**Conclusions:**

As anticipated, PT/OT utilization and burn center participation declined over time. However, patients receiving burn center services showed improved community integration. Despite slight benefits to burn center rehabilitation, cross-sectional analysis found no significant differences in outcomes, indicating that therapy can be effectively delivered in non-burn center settings. Future studies comparing functional outcomes between those who receive outpatient therapy and those who do not may offer deeper insights.

**Applicability of Research to Practice:**

This study shows that early and sustained rehabilitation at burn centers can significantly aid social recovery, but all rehabilitation remains important.

**Funding for the Study:**

The contents of this abstract were developed under a grant from the National Institute on Disability, Independent Living, and Rehabilitation Research (NIDILRR grant number 90DPBU0007 and 90DPBU0008). NIDILRR is a Center within the Administration for Community Living (ACL), Department of health and Human Services (HHS). The contents of this abstract do not necessarily represent the policy of NIDILRR, ACL, or HHS, and you should not assume endorsement by the Federal Government.

